# The consequences of generative AI for online knowledge communities

**DOI:** 10.1038/s41598-024-61221-0

**Published:** 2024-05-06

**Authors:** Gordon Burtch, Dokyun Lee, Zhichen Chen

**Affiliations:** Questrom School of Business, Boston University, Boston, MA 02215 USA

**Keywords:** Human behaviour, Psychology and behaviour

## Abstract

Generative artificial intelligence technologies, especially large language models (LLMs) like ChatGPT, are revolutionizing information acquisition and content production across a variety of domains. These technologies have a significant potential to impact participation and content production in online knowledge communities. We provide initial evidence of this, analyzing data from Stack Overflow and Reddit developer communities between October 2021 and March 2023, documenting ChatGPT’s influence on user activity in the former. We observe significant declines in both website visits and question volumes at Stack Overflow, particularly around topics where ChatGPT excels. By contrast, activity in Reddit communities shows no evidence of decline, suggesting the importance of social fabric as a buffer against the community-degrading effects of LLMs. Finally, the decline in participation on Stack Overflow is found to be concentrated among newer users, indicating that more junior, less socially embedded users are particularly likely to exit.

## Introduction

Recent advancements in generative artificial intelligence (Gen AI) technologies, especially large language models (LLMs) such as ChatGPT, have been significant. LLMs demonstrate remarkable proficiency in tasks that involve information retrieval and content creation^1–3^. Given these capabilities, it is important to consider their potential to drive seismic shifts in the way knowledge is developed and exchanged within online knowledge communities^4,5^.

LLMs may drive both positive and negative impacts on participation and activity at online knowledge communities. On the positive side, LLMs can enhance knowledge sharing by providing immediate, relevant responses to user queries, potentially bolstering community engagement by helping users to efficiently address a wider range of peer questions. Viewed from this perspective, Gen AI tools may complement and enhance existing activities in a community, enabling a greater supply of information. On the negative side, LLMs may replace online knowledge communities altogether.

If the displacement effect dominates, it would give rise to several serious concerns. First, while LLMs offer innovative solutions for information retrieval and content creation and have been shown to significantly enhance individual productivity in a variety of writing and coding tasks, they have also been found to hallucinate, i.e., providing ‘confidently incorrect’ responses to user queries^6^, and to undermine worker performance on certain types of tasks^3^*.* Second, if individual participation in online communities were to decline, this would imply a decline in opportunities for all manner of interpersonal interaction, upon which many important activities depend, e.g., collaboration, mentorship, job search. Further, to the extent a similar dynamic may emerge within formal organizations and work contexts, it would raise the prospect of analogous declines in organizational attachment, peer learning, career advancement and innovation^7–12^.

With the above in mind, we address two questions in this work. First, we examine the effects that generative artificial intelligence (AI), particularly large language models (LLMs), have on individual engagement in online knowledge communities. Specifically, we assess how LLMs influence user participation and content creation in online knowledge communities. Second, we explore factors that moderate (amplify or attenuate) the effects of LLMs on participation and content creation at online knowledge communities. By addressing these relationships, we aim to advance our understanding of the role LLMs may play in shaping the future of knowledge sharing and collaboration online. Further, we seek to provide insights into approaches and strategies that can encourage a sustainable knowledge sharing dynamic between human users and AI technologies.

We evaluate our questions in the context of ChatGPT’s release, in late November of 2022. We start by examining how the release of ChatGPT impacted Stack Overflow. We show that ChatGPT’s release led to a marked decline in web traffic to Stack Overflow, and a commensurate decline in question posting volumes. We then consider how declines in participation may vary across community contexts. Leveraging data on posting activity in Reddit developer communities over the same period, we highlight a notable contrast: no detectible declines in participation. We attribute this difference to social fabric; whereas Stock Overflow focuses on pure information exchange, Reddit developer communities are characterized by stronger social bonds. Further, considering heterogeneity across topic domains within Stack Overflow, we show that declines in participation varied greatly depending on the availability of historical community data, a likely proxy for LLM’s ability to address questions in a domain, given that data would likely have been used in training. Finally, we explore which users were most affected by ChatGPT’s release, and the impact ChatGPT has had on the characteristics of content being posted. We show that newer users were most likely to exit the community after ChatGPT was released. Further, and relatedly, we show that the questions posted to Stack Overflow became systematically more complex and sophisticated after ChatGPT’s release.

## Methods

To address these questions, we leverage a combination of data sources and methods (additional details are provided in the supplement). First, we employ a proprietary dataset capturing daily aggregate counts of visitors to stackoverflow.com, and a large set of other popular websites. This data covers the period from September 2022 through March 2023. Additionally, we employ data on the questions and answers posted to Stack Overflow, along with characteristics of the posting users, from two calendar periods that cover the same span of the calendar year. The two samples cover October 2021 through mid-March of 2022, and October 2022 through mid-March of 2023. These data sets were obtained via the Stack Exchange Data Explorer, which provides downloadable, anonymized data on activity in different Stack Exchange communities. Further, we employ data from subredditstats.com, which tracks aggregate daily counts of posting volumes to each sub-Reddit. Our data sources do not include any personal user information, and none of our analyses make use of any personal user information.

We first examined the effect that ChatGPT’s release on November 30th of 2022 had on web traffic arriving at Stack Overflow, leveraging the daily web traffic dataset. The sample, sourced from SimilarWeb, includes daily traffic to the top 1000 websites. We employ a variant of the synthetic control method^13^, namely Synthetic Control Using LASSO, or SCUL^14^. Taking the time series of web visits to stackoverflow.com as treated, the method identifies, via LASSO^15^, a linear, weighted combination of candidate control series (websites) that yields an accurate prediction of traffic to stackoverflow.com prior to ChatGPT’s release. The resulting linear combination is then used to impute a counterfactual estimate of traffic at stackoverflow.com in the period following ChatGPT’s release, reflecting predictions of web traffic volumes that would have been observed in the absence of ChatGPT.

Second, we examined ChatGPT’s effects on the volume of questions being posted to Stack Overflow. We identified the top 50 most popular topic tags associated with questions on Stack Overflow during our period of study, calculating the daily count of questions including each tag over a time window bracketing the date of ChatGPT’s release. We then followed the approach of Refs.^16,17^, constructing the same set of topic panels for the same calendar period, one year prior, to serve as our control within a difference-in-differences design, to estimate an average treatment effect, and to enable evaluation both of the parallel trends assumption (which is supported by the absence of significant pre-treatment differences) and treatment effect dynamics^18^. Figure S1 in the supplement provides a visual explanation of our research design.

Third, we considered whether the effects might differ across online knowledge communities, depending on the degree to which a community is focused strictly on information exchange. That is, we considered the potential mitigating effect of social fabric, i.e. social bonds and connections, as a buffer against LLMs negative effects on connection with human peers. The logic for this test is that LLMs, despite being capable of high-quality information provision around many topics, are of less clear value as a pure substitute for human social connections^19^. We thus contrasted our average effect estimates from Stack Overflow with effect estimates obtained using panels of daily posting volumes from analogous sub-communities at Reddit (sub-Reddits), focused on the same sets of topics. Reddit is a useful point of comparison because it has been well documented that Reddit developer communities are relatively more social and communal than Stack Overflow^20,21^. We also explored heterogeneity in the Stack Overflow effects across topics, repeating our difference-in-differences regression for each Stack Overflow topic and associated sub-reddit.

Lastly, we explored shifts in the average characteristics of users and questions at Stack Overflow following ChatGPT’s release, specifically in terms of the posting users’ account tenure, in days, and, relatedly, the average complexity of posted questions. It is reasonable to expect that the individuals most likely to rely on ChatGPT are junior, newer members of the community, as these individuals likely have less social attachment to the community, and they are likely to ask relatively simpler questions, which ChatGPT is better able to address. In turn, it is reasonable to expect that the questions that fail to be posted are those that would have been relatively simpler. We tested these possibilities in two ways, considering question-level data from Stack Overflow. We began by estimating the effect of ChatGPT’s release on the average tenure (in days) of posting users’ accounts. Next, we estimated a similar model, considering the average frequency of ‘long’ words (words with 6 or more characters) within posted questions, as a proxy for complexity.

## Results

### Overall impact of LLMs on community engagement

**Figure 1:**
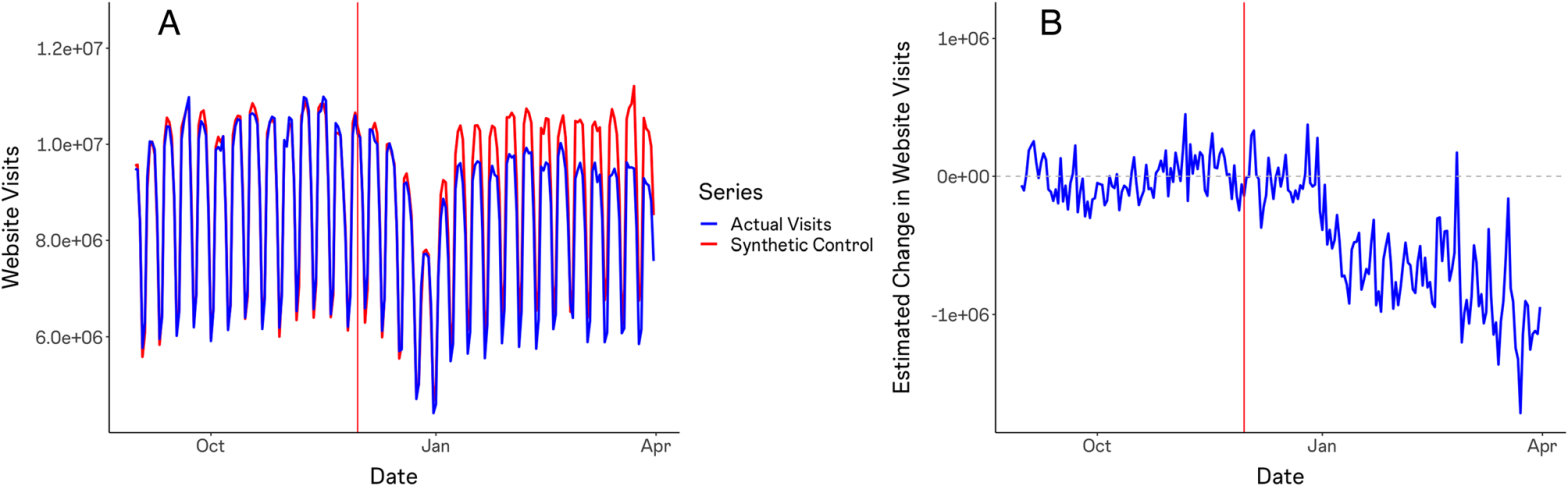
Synthetic control estimates of decline in daily web traffic to stack overflow. Estimates are obtained via synthetic control using LASSO (SCUL), based on daily web traffic estimates according to SimilarWeb for the 1000 most popular websites on the internet. Panel (**A**) depicts the actual web traffic volumes (in blue) recorded by SimilarWeb alongside the Synthetic Control (in red). Panel (**B**) depicts the difference between the two series, reflecting the estimated causal effect of ChatGPT.

Figure 1A depicts the actual daily web traffic to Stack Overflow (blue) alongside our estimates of the traffic that Stack Overflow would have experienced in the absence of ChatGPT’s release (red). The Synthetic Control estimates closely mirror the true time series prior to ChatGPT’s release, supporting their validity as a counterfactual for what would have occurred post. Figure 1B presents the difference between these time series. We estimate that Stack Overflow’s daily web traffic has declined by approximately 1 million individuals per day, equivalent to approximately 12% of the site’s daily web traffic just prior to ChatGPT’s release.

### LLMs' effect on user content production

**Figure 2:**
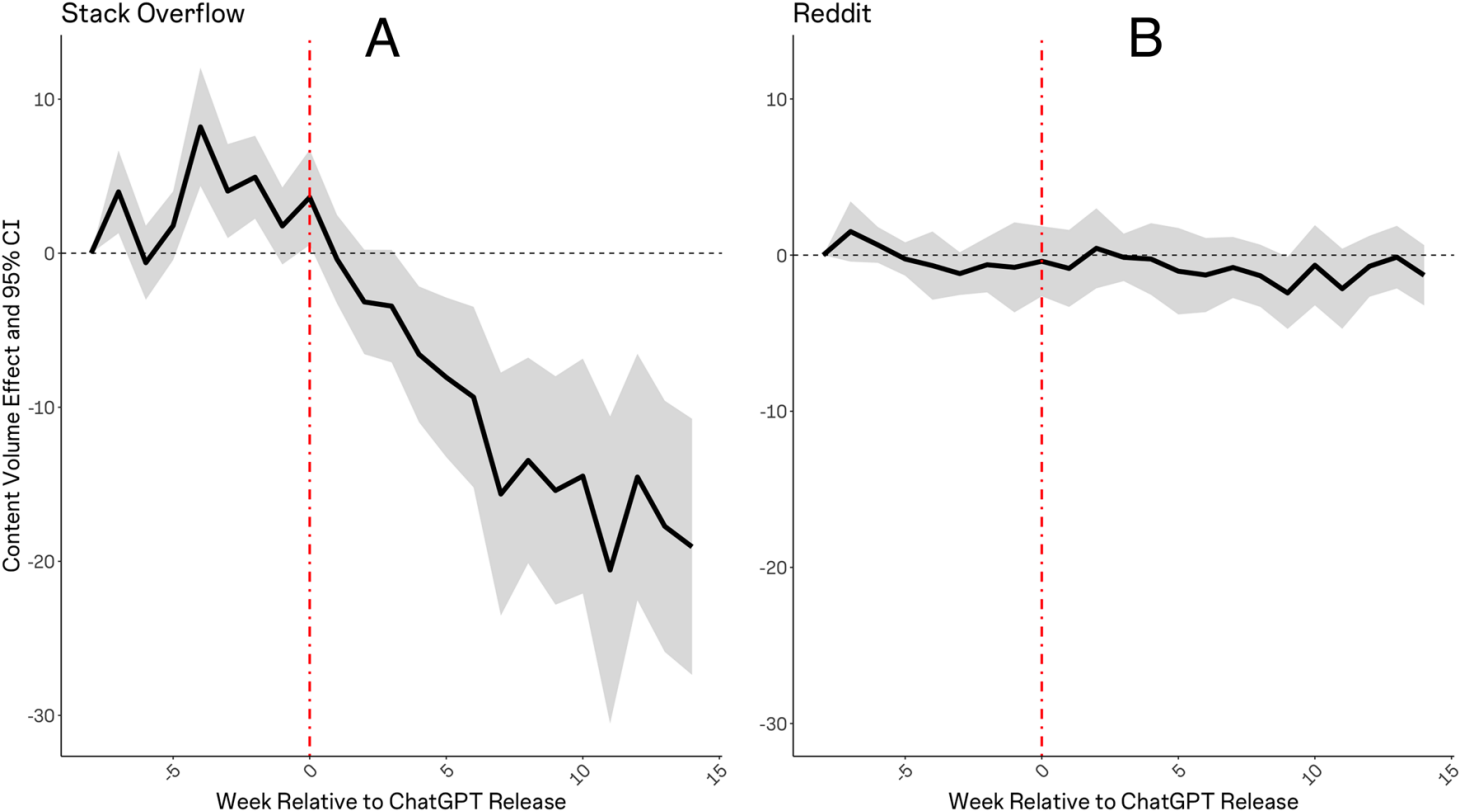
Estimated effects of ChatGPT on user activity at stack overflow and reddit. Estimates are obtained via difference-in-differences regression, comparing content posting volumes over a period bracketing the release of ChatGPT (on November 30th, 2022) with a window of equal length observed one calendar year prior. Panel (**A**) depicts effects over time (by week) on Stack Overflow question volumes per topic. Panel (**B**) depicts effects on Reddit posting volumes, per sub-reddit, for sub-reddits dealing with an overlapping set of topics. The shaded area represents 95% confidence intervals.

Our difference-in-differences estimations employing data on posting activity at Stack Overflow revealed that question posting volumes per-topic on Stack Overflow have declined markedly since ChatGPT’s release (Fig. 2A). This result reinforces the idea that LLMs are replacing online communities as a source of knowledge for many users. Repeating the same analysis using Reddit data, we observed no evidence that ChatGPT has had any effects on user engagement at Reddit (Fig. 2B). We replicate these results in Fig. S2 of the supplement employing the matrix completion estimator of Ref.^22^.

### Heterogeneity in ChatGPT’s effect on stack overflow posting volumes by topic

**Figure 3:**
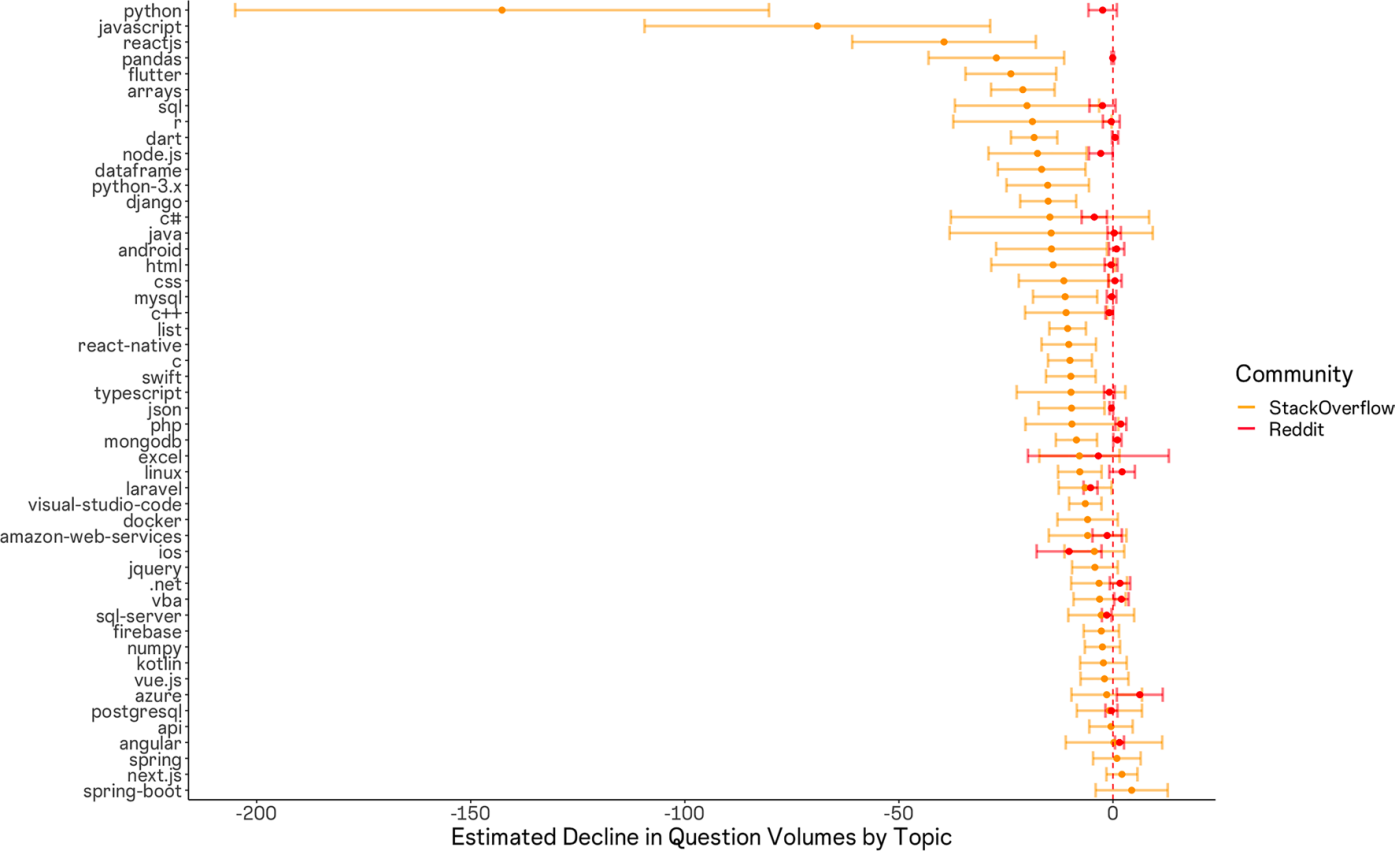
Topic-specific effects of ChatGPT on stack overflow and reddit. Estimates are obtained via difference-in-differences regression, per topic. The figure depicts effect estimates for each stack overflow topic (in orange) with 95% confidence intervals and estimates for each sub-reddit (in red), where available. Note that data on sub-reddit posting volumes was not available for three sub-reddit communities: javascript, jQuery, and Django. Other Reddit estimates are omitted due to the lack of a clearly analogous sub-reddit addressing that topic.

We observed a great deal of heterogeneity across Stack Overflow topics, yet consistently null results across sub-reddits (Fig. 3). Our estimates thus indicate, again, that Reddit developer communities have been largely unaffected by ChatGPT’s release. Our Stack Overflow results further indicate that the most substantially affected topics are those most heavily tied to concrete, self-contained software coding activities. That is, the most heavily affected topics are also those where we might anticipate that ChatGPT would perform quite well, due to the prevalence of accessible training data.

For example, Python, CSS, Flutter, ReactJS, Django, SQL, Arrays, and Pandas are all references to programming languages, specific programming libraries, or data types and structures that one might encounter while working with a programming language. In contrast, relatively unaffected tags appear more likely to relate to topics involving complex tasks, requiring not only appropriate syntax but also contextual information that would often have been outside of the scope of ChatGPT's training data. For example, Spring and Spring-boot are Java-based frameworks for enterprise solutions, often involving back-end (server-side) programming logic with private enterprise knowledge bases and software infrastructures. Questions related to these topics are intuitive questions for which an automated (i.e. cut-and-paste) solution would be less straightforward, and less likely to appear in the textual training data available for training the LLM. Additional examples here include the tags related to Amazon Web Services, Firebase, Docker, SQL Server, and Microsoft Azure.

**Figure 4:**
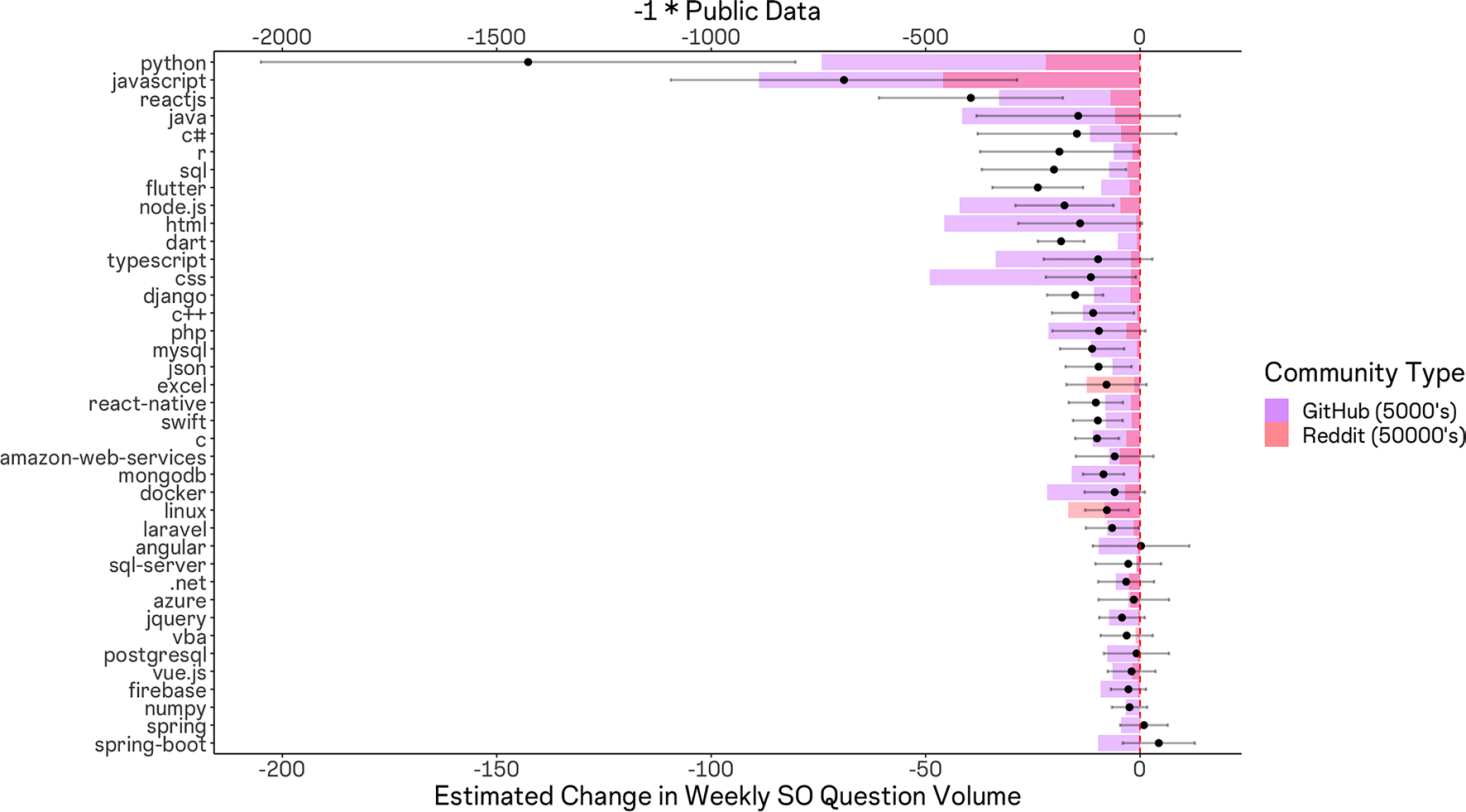
Topic-specific effects of ChatGPT on stack overflow (black points with 95% confidence intervals) with Number of Github repositories (purple) and sub-reddit subscribers (red) overlaid. We observe a rough correlation between the volume of Github repositories making use of a given language or framework, the level of activity in associated sub-reddit communities, and the magnitude of effect sizes. This associate suggests effects are larger for topics where more public data was available to train the LLM.

To evaluate this possible explanation more directly, we collected data on the volume of active GitHub repositories making use of each language or framework, as well as the number of individuals subscribed to sub-reddits focused on each language or framework. We then plotted a scaled measure of each value atop the observed effect sizes and obtained Fig. 4. The figure indicates a rough correlation between available public sources of training data and our effect sizes.

### ChatGPT’s effect on average user account age and question complexity

**Figure 5:**
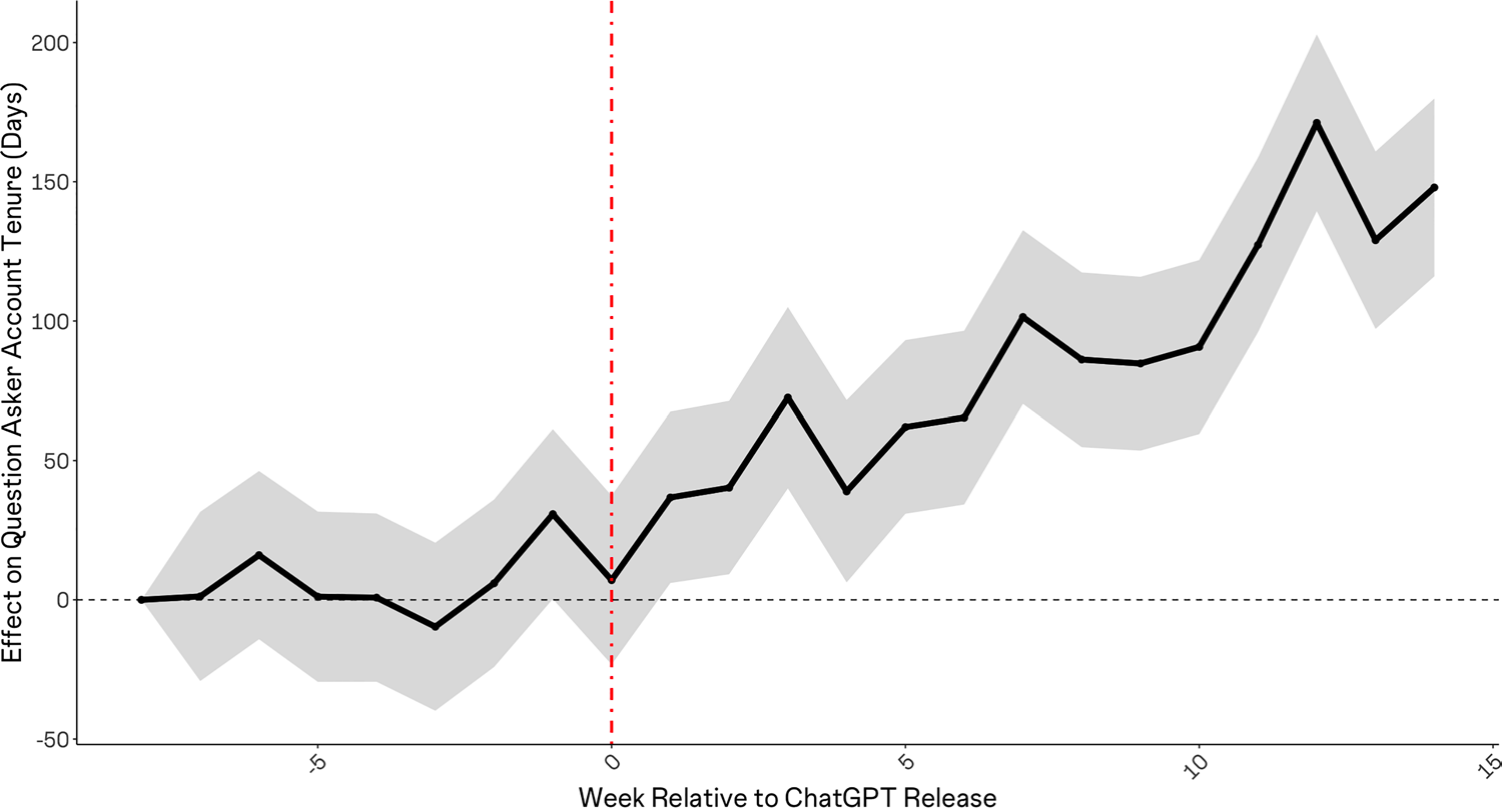
Effect of ChatGPT release on the average tenure (in days) of user accounts posting questions to stack overflow. Shortly after ChatGPT’s release, we see a systematic rise in the average age (in days) for the user accounts posting questions to StackOverflow. We see that average account age rises systematically once ChatGPT is released, consistent with newer accounts systematically reducing their participation and exiting the community.

**Figure 6:**
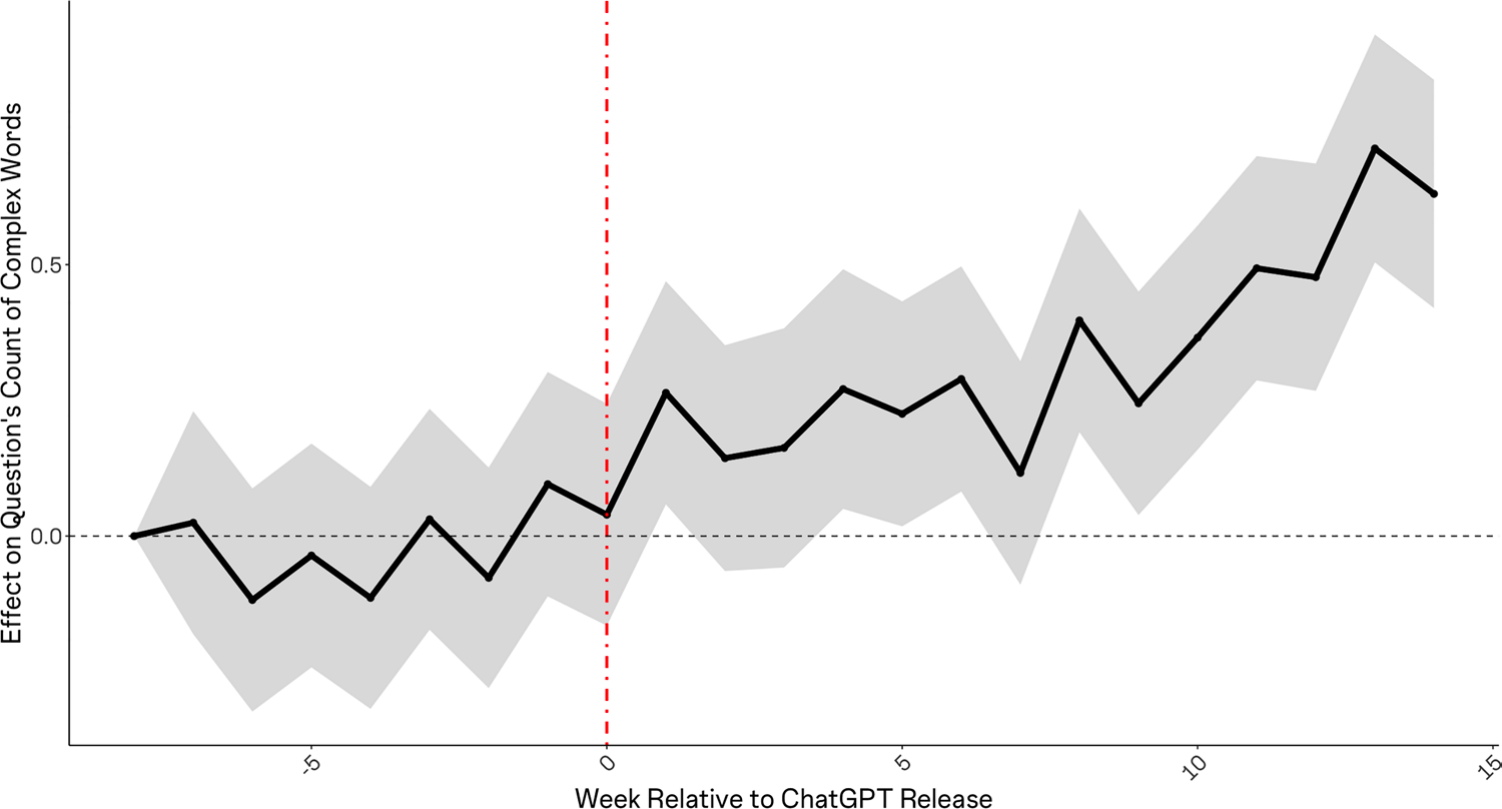
Effect of ChatGPT's release on the average complexity of questions posted to Stack Overflow, reflected by the average frequency of ‘long’ words (words with 6 or more characters). Shortly after ChatGPT’s release, we see a systematic rise in the average complexity of questions. This result is again consistent with the idea that newer accounts systematically reduced their participation and exited the community.

Figure 5 depicts the change in average posting users’ account tenure, making clear that, upon ChatGPT’s release, a systematic rise began to take place, such that users were increasingly likely to be more established, older accounts. The implication of this result is that newer user accounts became systematically less likely to participate in the Stack Overflow community after ChatGPT became available. Figure 6 depicts the effects, indicating that questions exhibited a systematic rise in complexity following the release of ChatGPT.

These findings, consistent with the idea that more junior and less experienced users began to exit might be cause for concern if a similar dynamic is playing out in more formal organization and work contexts. This is because junior individuals may stand to lose the most from declines in peer interaction—these individuals typically are more marginal members of organizations and thus have less robust networks and have the most to lose in terms of opportunities for career advancement^23^. Further, these individuals may be least capable of recognizing mistakes in the output of LLMs, which are well known to engage in hallucination, providing ‘confidently wrong’ answers to user queries^6^. Indeed, recent work observes that non-experts face the greatest difficulty determining whether the information they have obtained from an LLM is correct^24^.

## Discussion

We have shown that ChatGPTs release was associated with a discontinuous decline in web traffic and question posting volumes at Stock Overflow. This result is consistent with the idea that many individuals are now relying on LLMs for knowledge acquisition in lieu of human peers in online knowledge communities. Our results demonstrate that these effects manifested for Stack Overflow, yet not for Reddit developer communities.

Further, we have shown that these effects were more pronounced for very popular topics as compared to less popular topics, and evidence suggests that this heterogeneity derived from the volume of training data available for LLM training prior to ChatGPTs release. Finally, our results demonstrate that ChatGPT’s release was associated with a significance, discontinuous increase in the average tenure of accounts participating on Stack Overflow, and in the complexity of questions posted (as reflected by the prevalence of lengthy words within questions). These results are consistent with the idea that that newer, less expert users were more likely to begin relying on ChatGPT in lieu of the online knowledge community.

Our findings bear several important implications for the management of online knowledge communities. For online communities, our findings highlight the importance of social fabric as a means of ensuring the sustainability and success of online communities in the age of generative AI. Our findings thus highlight that managers of online knowledge communities can combat the eroding influence of LLMs by enabling socialization, as a complement to pure information exchange. Our findings also highlight how content characteristics and community membership can shift because of LLMs, observations that can inform community managers content moderation strategies and their activities centered on community growth and churn prevention.

Beyond the potential concerns about what the observed dynamics may imply for online communities and their members, our findings also raise important concerns about the future of content production in online communities, which by all accounts have served as a key source of training data for many of the most popular LLMs, including OpenAI’s GPT. To the extent content production declines in these open communities, it will reinforce concerns that have been raised in the literature about limitations on the volume of data available for model training^25^. Our findings suggest that long-term content licensing agreements that have recently been signed between LLM creators and online community operators may be undermined. If these issues are left unaddressed, the continued advancement of generative AI models may necessitate that their creators identify alternative data sources.

## Conclusion

Our work is not without limitations, some of which present opportunities for future research. First, for our research design to yield causal interpretations, we must assume the absence of confounded treatments. For example, were another large online community to have emerged around the same time, the possibility exists that it may explain the decline in participation at Stack Overflow. Second, our study lacks a nuanced analysis of changes in content characteristics. Although we study changes in answer quality using net vote scores (see the supplement), our measures may reflect changes in other aspects unrelated to information quality. Similarly, although we study changes in question complexity, our measure of complexity is tied to word length. Future work can thus revisit these questions employing a variety of other measures of quality and complexity.

Third, although we have shown a decline in participation at Stack Overflow, we are unable to speak to whether the same dynamic is playing out in other organizational settings, e.g. workplaces. It is also important to recognize that the context of our analyses may be unique. To the extent Stack Overflow and Reddit developer communities might not be representative of developer communities more broadly, the generalizability of these results would be constrained. Relatedly, it is possible that the results we observe are unique to knowledge communities that focus on software development and information technology. The dynamics of content production may differ markedly in other knowledge domains. Finally, our work demonstrates effects over a relatively short period of time (several months). It is possible that the longer-run dynamics of the observed effects may shift. Given these points, future work can and should endeavor to explore the generalizability of our findings to other communities, and future work should examine the longer-run effects of generative AI technologies on community participation and knowledge sharing.

We anticipate that our study will inspire more sophisticated analyses of the effects that generative AI technologies, including LLMs, but also generative image, audio, and video models, may have on patterns of knowledge sharing and collaboration within organizations and society more broadly. Such work is crucially needed, to better understand the nuances of where and when individuals may rely on human peers versus Generative AI tools, and the desirable and undesirable consequences for organizations and society, such that we can begin to plan for and manage this new dynamic.

### Supplementary Information

Supplementary Information.

## Data Availability

Data on Stack Overflow users, questions, and answers was obtained via the Stack Exchange Data Explorer at https://data.stackexchange.com/stackoverflow/query/new. Data on sub-reddit posting volumes was obtained from https://subredditstats.com. Similar Web daily web traffic data is not available for public dissemination, though it is available for purchase from https://deweydata.io. Stack Overflow data, Reddit data and analysis scripts are available in a public repository at the OSF: https://osf.io/qs6b3/.
