# Supplementary material for: The consequences of generative AI for online knowledge communities
Source: Sci Rep. 2024 May 6;14:10413. doi: 10.1038/s41598-024-61221-0 (PMC11074245; doi:10.1038/s41598-024-61221-0)
Supplement: Supplementary file 1 — Supplementary Information. [file 41598_2024_61221_MOESM1_ESM.pdf]

Supplementary Materials for

The Consequences of Generative AI for Online Knowledge Communities

Gordon Burtch, Dokyun Lee, Zhichen Chen

Corresponding author: gburtch@bu.edu

The PDF file includes:

Materials and Methods
Supplementary Text
Figs. S1 to S3
Tables S1 to S2

Other Supplementary Materials for this manuscript include the following:

None

Materials and Methods

Data Availability Statement

The datasets (or portions thereof) generated and/or analysed during the current study are not publicly available due to third-party licensing agreements but are available from the corresponding author on reasonable request.

Replication scripts for all analyses have been made available in a public repository at the OSF: https://osf.io/qs6b3/. The repository contains all publicly available data. Data used to estimate ChatGPT's effects on Stack Overflow web traffic are not publicly available, as it is subject to a third-party licensing agreement. That data can be purchased from DeweyData.io: https://www.deweydata.io/data-partners/similarweb.

Synthetic Control Using LASSO (SCUL)

SCUL (*14*) employs LASSO (*15*) to learn a single treated panel's pre-intervention outcomes, selecting a linear weighted combination of control panels' pre-intervention outcomes. The resulting model is then used to project the counterfactual for the treated panel into the post-treatment period. In our case, the treated panel is the time series of daily web traffic associated with stackoverflow.com, and candidate controls that LASSO draws from include the other 999 traffic series. The date of treatment is November 30th of 2022, when ChatGPT was officially released.

Research Design for Question- & Post-volume Effects

Our research design is depicted graphically in Fig. S1., an approach that parallels that of (*16,17*). We use the timing of ChatGPT's release, on November 30th, 2022, as an event study. ChatGPT's release affects all individuals and online communities at the same time, which poses a challenge for selecting a control group. Our selected control set is thus activity occurring within the same set of topics and sub-reddits one year prior. This choice of control has the benefit of capturing any seasonal patterns in contributions by community topic. Seasonal patterns are likely here, given the topics are frequently technical in nature and tied to regular work activities.

Using this control, we estimate a difference-in-differences specification as reflected by Equation (1), where *i* indexes topics or sub-reddits, *c* indexes days relative to November 30th, and *t* indexes whether an observation pertains to the time window bracketing ChatGPT's actual release, versus the year prior. Further, *Volume* is our outcome measure, either question volumes or post volumes on Reddit, *Treat* is an indicator of whether data pertains to the period around ChatGPT's release (versus the year prior), and *Post* is also a binary indicator, of whether an observation takes place after November 30th versus before. Our specification further includes topic (sub-reddit) fixed effects, *Topic*, and week (relative to November 30th) fixed effects, *Week* Finally, *e* is an idiosyncratic error term. When performing the estimation, we cluster standard errors by topic (sub-reddit). Regression estimates for these average effects are reported for Stack Overflow and Reddit in Table S1.

$$Volume_{i,c,t} = Treat_t + Treat_t \cdot Post_c + Topic_i + Week_c + e_{i,c,t} \qquad (1)$$

Beyond this focal estimation, we also consider Equation 2, estimating a variant of the above model that expands the Post dummy into a vector of dummies reflecting an observation's chronological distance from November 30th, in weeks (*RelWeek*). In this estimation, we omit the earliest calendar week dummy (-8), taking it as reference. In each model, we again incorporate a topic (sub-reddit) fixed effect, and we cluster our standard errors by topic (sub-reddit).

$$Volume_{i,c,t} = Treat_t + Treat_t \cdot RelWeek_c + Topic_i + W_c + e_{i,c,t} \quad (2)$$

This dynamic specification is useful because it enables a joint test of i) dynamics of the treatment effect post treatment, and ii) the parallel trend assumption. The latter test is facilitated by examining the pre-treatment effect estimates. Under the assumption of parallel trends, we would expect no significant differences prior to the onset of treatment, as this would be consistent with the idea that the treatment and control groups exhibited parallel trends in their outcomes prior to the onset of treatment, and thus that the control group can serve as a reasonable counterfactual for what would have happened to the treated group in the absence of treatment. This test is particularly important to consider in our Stack Overflow analyses, because Stack Overflow was reported to have been losing some of its user traffic even prior to the release of ChatGPT, which raises the potential that the treatment group had begun to deviate from the control prior to ChatGPTs release. Fortunately, despite this, our various dynamic estimations reveal no strong evidence that the parallel trend assumption is violated.

<u>Account Age & Question Complexity Effects</u>

To estimate the effect of ChatGPT's release on the average tenure (in days) of user accounts posting questions to Stack Overflow, as well as the average complexity of questions (based on the frequency of words containing 6 or more characters), we employ our question-level dataset and the same research design depicted in Figure S1. The specification is relatively simpler, however, as we have a single observation for each unit of analysis, i.e., questions. Our outcomes of interest are the account tenure, in days, *Age,* of the question author at the time a question is posted, and the volume of words with 6 or more characters that appears in the question, our proxy for *Complexity*. We regress these outcomes on the interaction between our indicator or treatment, *Treat*, and our *RelWeek* dummies, as before. We further include a vector of week (relative to November 30th) fixed effects. Our estimations are captured by Equation 3, below, where *c* again indexes calendar time and *t* again indexes whether an observation relates to the period bracketing ChatGPT's actual release, or the control period one year prior.

$$Age\,/\,Complexity_{c,t} = Treat_t + Treat_t \cdot RelWeek_c + W_c + e_{c,t} \quad (3)$$

Supplementary Text

<u>Matrix Completion Estimates</u>

We repeated our primary analyses of the dynamic effects of ChatGPT release on question- and post-volumes at Stack Overflow and Reddit, respectively, employing an alternative estimation technique, namely the matrix completion estimator of *(22)*. As this estimator requires that

observations be uniquely indexed by a panel identifier and time identifier, we aggregate the question and posting data by combinations of *RelWeek* and *Treat*, treating weeks of activity for a given topic in the control period as distinct from the same panels observed in the treatment period. Thus, for Python, we construct two panels, one occurring over the weeks surrounding November 30th of 2021 (control), and a second surrounding November 30th of 2022 (treated). We then implement the matrix completion estimator, indexing the estimation based on panel identifier and an integer reflecting the sequence of weekly observations in each panel. Doing so, we obtained the results depicted in Figure S2.

<u>Answer Quality Effects</u>

Finally, we also conducted an analysis that sought to understand whether ChatGPT's effects have led to meaningful declines in the quality of answers appearing on Stack Overflow. To conduct this analysis, we employ our answer-level dataset. We relied on the same identification strategy, at the answer level. We estimated shifts in average peer voting outcomes associated with posted answers. Recognizing that a decline in web traffic to Stack Overflow may mean that there are fewer individuals present to cast their votes, we would expect vote totals to contract toward zero regardless of any changes in answer quality. To account for any possible role of declines in the voting population, we focus on answers that receive at least one vote and we model the probability that the net of votes received bears a negative (rather than positive) sign. Admittedly, this measure still has limitations, as it is possible that votes may shift for other reasons, unrelated to answer quality, e.g., answer structure may have changed, in ways that deviated from community norms, for example, if responding users were making use of ChatGPT to craft answers. Bearing that limitation in mind, we estimate a regression of the form expressed in Equation 4, where, q indexes the question being answered, c indexes calendar time (relative to November 30th), and t again indexes whether the data pertains to the window of time bracketing ChatGPT's release (November 30th of 2022) or the year prior. We once again incorporate a relative (calendar) week fixed effect, W, and a question fixed effect, Q. The latter implies that our estimations will exploit variation in answer timing conditional on the question being answered. This also ensures that the estimation contrasts vote outcomes between answers to the exact same question.

$$NetNegative_{q,c,t} = Treat_t + Treat_t \cdot Post_c + Q_q + W_c + e_{q,c,t} \quad (4)$$

Subsequently, we extend the regression equation, obtaining Equation 5, where we incorporate a responding-user fixed effect, γ. We thus introduce an additional subscript, u, reflecting a responding user index. By contrasting the estimates from Equations 4 and 5, we can understand whether any estimated changes in answer quality are attributable to the exit of users versus a decline in answer quality within users, e.g., due to a reliance on ChatGPT. Our outcome of interest is a binary indicator of whether the net vote score is negative, *NetNegative*. In addition to the simple average effect, we subsequently examine a dynamic specification.

$$NetNegative_{q,u,c,t} = Treat_t + Post_c + Treat_t \cdot Post_c + Q_q + W_c + \gamma_u + \mu_{q,u,c,t} \quad (5)$$

We report the results of our average effect estimates in Table S2, without and with a responding user fixed effect. Further, we depict our dynamic estimates graphically, in Figure S3.

Our first estimation, absent a responding user fixed effect, indicates that the probability an answer is net downvoted rises systematically after ChatGPT is released, and the dynamic estimates tell a similar story (Fig. S3). Once we incorporate a responding user fixed effect, however, the results disappear. These estimates collectively indicate that answer quality is indeed degrading, but that this is not due to individuals' use of ChatGPT when responding to peers' questions; rather, the decline in answer quality appears due to user exit. As the community shrinks, the efficacy of the crowdsourcing process degrades, with a reduced likelihood of long-tail, extreme value solutions to posted queries.

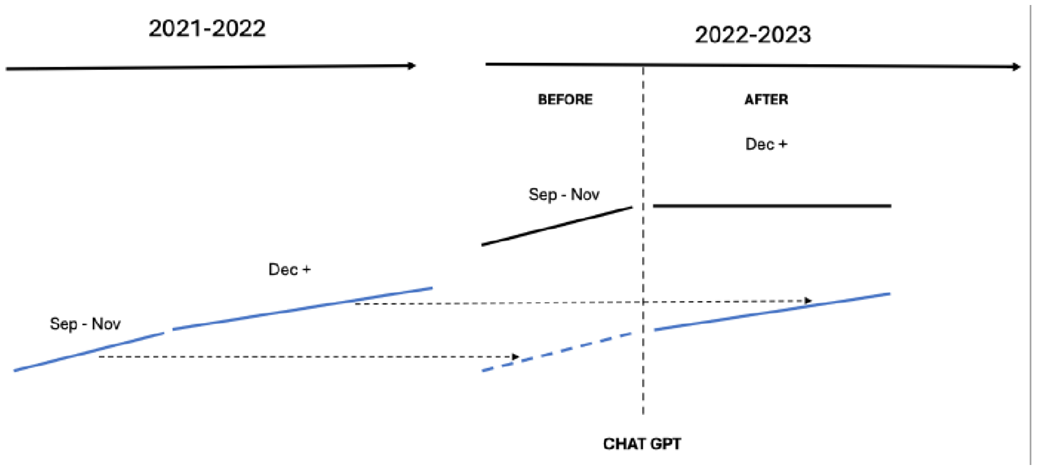

Fig. S1.
A graphical depiction of the research design we employ to estimate the effect of ChatGPT on the daily volume of postings per-topic (per sub-reddit) at Stack Overflow (Reddit).

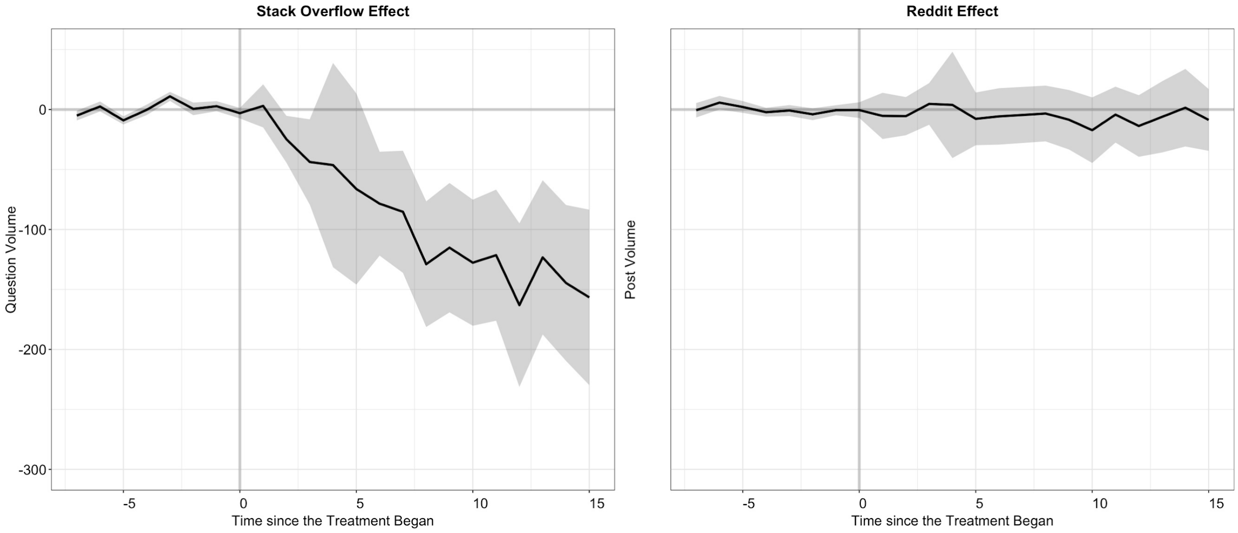

Fig. S2.

Matrix completion estimates of the effect ChatGPT has had on average weekly question volumes per topic, and average weekly post volumes per sub-Reddit. We observe consistent results; ChatGPT has reduced posts to Stack Overflow (left) but has had no discernible effect on Reddit (right). Error bands reflect 95% confidence intervals based on bootstrapped standard errors, clustered by topic, with 200 bootstrap iterations.

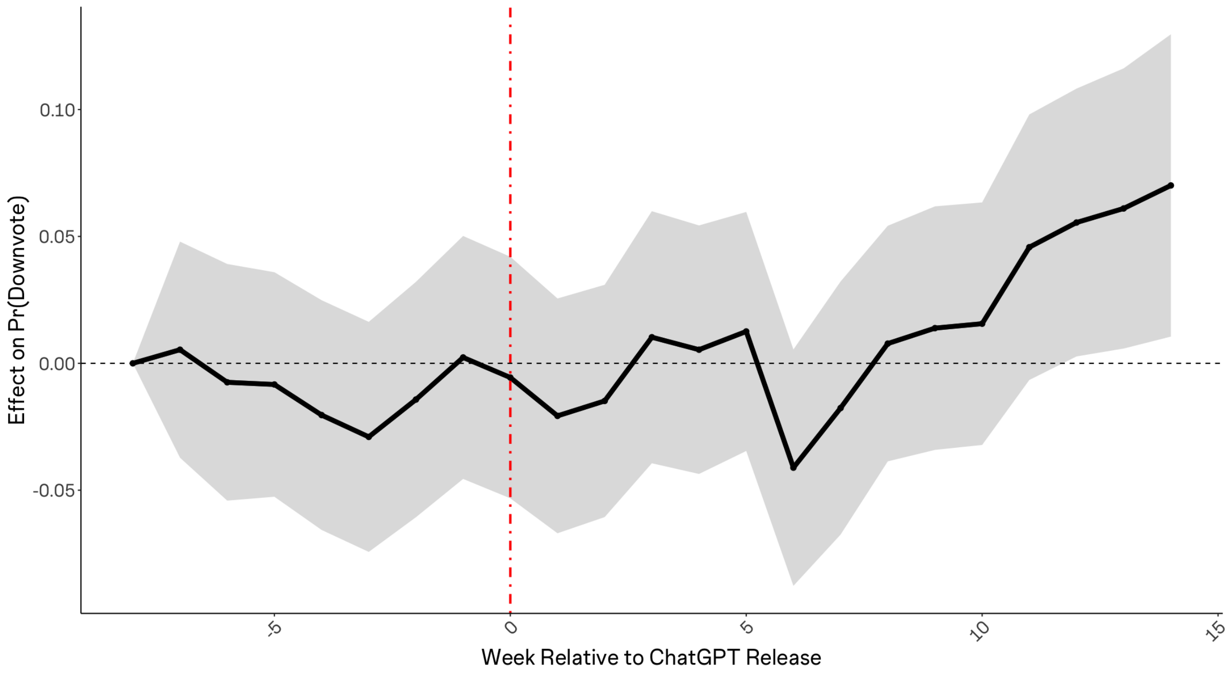

Fig. S3.
Dynamic effect of ChatGPT's release on the probability that answers to Stack Overflow questions that receive at least one vote from the community are net downvoted. Results demonstrate that the probability of a downvote taking place increases after ChatGPT is released, indicating a decline in the quality of answers. The error bands reflect 95% confidence intervals based on cluster robust standard errors, clustered by question.

	Stack Overflow	Reddit
Treat	9.244***	1.830
	(2.094)	(1.403)
Treat × Post	-13.554***	-0.701
	(3.117)	(0.596)
Observations	16,100	8,316
R2 Adj.	0.908	0.844
FE: Topic	Yes	Yes
FE: Year-Week	Yes	Yes

Notes:*(1) + $p < 0.1$, * $p < 0.05$, ** $p < 0.01$, *** $p < 0.001$*
(2) Standard errors in parentheses, clustered by topic.
(3) We are able to identify sub-reddits for approximately 50% of topics.

Table S1.
The average effect of ChatGPT's release on daily, per topic, question-posting volumes at Stack Overflow and daily per sub-reddit posting volumes at Reddit. We observe a statistically significant decline in daily question volumes per topic tag, on average, at Stack Overflow. By contrast, though we observe a negative coefficient in the case of Reddit, the estimate is not statistically significant. The estimate associated with Stack Overflow suggests an average daily decline of approximately 14 questions per topic.

	(No User FE)	(User FE)
Treat	0.035***	0.015
	(0.007)	(0.030)
Treat × Post	0.017*	-0.005
	(0.009)	(0.032)
Observations	710,509	710,509
R2	0.846	0.972
FE: Question	Yes	Yes
FE: Calendar Week	Yes	Yes
FE: User	No	Yes

Notes:+ $p < 0.1$, * $p < 0.05$, ** $p < 0.01$, *** $p < 0.001$
Standard errors in parentheses, clustered by question.

Table S2.

The average effect of ChatGPT's release on the probability that a Stack Overflow answer is net downvoted by the crowd. We focus on a binary indicator of net down-voting and we restrict the estimation sample to answers that received at least 1 vote from the community, to address the fact that declines in site traffic will drive a contraction of vote totals toward zero. We find a significant, positive effect on the probability that an answer receives a net downvote from the crowd absent a responding user fixed effect, but that result becomes insignificant in the presence of a responding user fixed effect. These findings indicate that the decline in answer quality is not within-user (i.e., due to the use of ChatGPT in answering questions); rather, it is due to which users are answering questions, implying that quality declines as users exit the community and fewer individuals respond to questions.
